# Supplementary material for: Transcription Factor VM1G_06867: A Requirement for Growth, Pathogenicity, Development, and Maintenance of Cell Wall Integrity in Valsa mali
Source: J Fungi (Basel). 2023 Jun 20;9(6):692. doi: 10.3390/jof9060692 (PMC10305253; doi:10.3390/jof9060692)
Supplement: Supplementary file 1 [file jof-09-00692-s001.zip › jof-2437077-supplementary.pdf]

Table S1. Primers used in this study

Primer name	Primer sequence (5' to 3')
06867-SY-F	GCTGCGGATGATTGCTTC
06867-SY-R	attcattgttgacctccactCAGGGCGTAATGAGATGC
06867-XY-F	gggcaaaggaatagagtagaCGAGGCACAACTGAACAC
06867-XY-R	GCGTAACAAAGGCTTAGAAC
HPH-F	AGTGGAGGTCAACAATGAAT
HPH-R	TCTACTCTATTCCTTTGCCC
06867-YZ-F1	AGAGCACGAAGGCGAACA
06867-YZ-R1	CCTACAGGGCGTAATGAGAT
06867-YZ-F2	TTCTTCACTTGGCACCTA
06867-YZ-R2	ACTGTGGTTTGCCTCTAT
HPH-YZ-F	TATTAGCAGACAGGAACGAGGAC
HPH-YZ-R	CTTCTGCGGGCGATTTGTGTA
06867 HB-F	actcactatagggcgaattgggtactcaaattggttACCGTCAAGGTCTCGTGC
06867 HB-R	caccaccccggtgaacagctcctcgcccttgctcacATATCGTAGCCTAGTTAATCGT

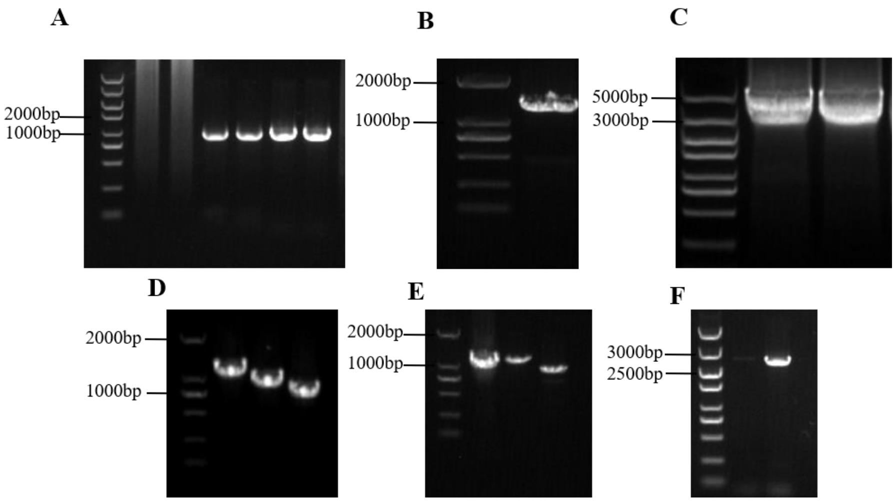

Figure S1. *VM1G_06867* gene replacement and complementarity. (A) Amplification of the upstream and downstream fragments. (B) Amplification of the hygromycin b phosphotransferase gene (hph) fragment using of primer pairs HPH-F and HPH-R. (C) Generation of the replacement fragment by double-join PCR. (D) PCR Screening and confirmation of the *VM1G_06867* single deletion mutant using the four primer pairs as described. (E) PCR screening and confirmation of the *VM1G_06867* double deletion mutant using the four primer pairs as described. (F) Generation of *VM1G_06867* fragments with their promoters using primer pairs 06867-HB-F/R.
